# Clinical significance of albumin to globulin ratio among patients with stroke-associated pneumonia

**DOI:** 10.3389/fnut.2022.970573

**Published:** 2022-08-16

**Authors:** Lingli Chen, Minjie Xu, Qiqi Huang, Yuntao Liu, Wenwei Ren

**Affiliations:** ^1^Department of Neurology, The First Affiliated Hospital of Wenzhou Medical University, Wenzhou, China; ^2^School of Mental Health, Wenzhou Medical University, Wenzhou, China; ^3^Department of Cardiac Care Unit, The First Affiliated Hospital of Wenzhou Medical University, Wenzhou, China

**Keywords:** albumin to globulin, acute ischemic stroke, pneumonia, predict, risk, ratio

## Abstract

**Background:**

It has been proven that the ratio of albumin to globulin (A/G) is a typical biomarker for monitoring inflammation and nutritional status. But the potential role of A/G in stroke-associated pneumonia (SAP) after acute ischemic stroke (AIS) remained unknown.

**Patients and methods:**

Following inclusion criteria, 5,173 AIS patients were included and segmented into SAP (*n* = 897) and non-SAP (*n* = 4,276) groups. The differences in variables between groups were compared. The logistic regression model was used to determine the association between A/G and SAP, and a forest plot was drawn.

**Results:**

Compared with the non-SAP group, the SAP group had a lower A/G level (*P* < 0.001). Then, A/G was divided into quartiles. In comparison to Q3 (A/G = 1.25–1.39), logistic regression revealed that patients with a lower A/G (A/G ≤ 1.09) had a higher risk of SAP (OR = 1.96, 95% CI, 1.56–2.46, *P* < 0.001). On the contrary, those with a higher A/G (A/G ≥ 1.4) had a lower SAP risk (OR = 0.73, 95% CI, 0.54–0.97, *P* = 0.029).

**Conclusion:**

The study revealed that a low A/G level was associated with an increased SAP risk. Appropriate preventative measures for SAP should be taken in AIS patients with a low A/G level.

## Introduction

Acute ischemic stroke (AIS), a life-threatening neurological disorder, is one of the leading causes of mortality and disability worldwide (1, 2). In patients with AIS, stroke-associated pneumonia (SAP) has been proven to be associated with a poor functional outcome and an increased risk of death (3). Thus, early identification of pneumonia risk factors may contribute to more effective SAP prevention and reduced mortality and disability.

Albumin to globulin ratio (A/G), a composite biomarker of two major serum elements, indicates inflammation and nutrition status (4). Different disorders, including cancer, infection, autoimmune disease, and stroke, are associated with A/G abnormalities (4–7). Prior research put forward A/G as an independent risk factor for survival in elderly individuals with chronic obstructive lung disease who had an acute exacerbation (8). A meta-analysis of 3,496 lung cancer patients indicated that those with a low A/G were more likely to have a worse prognosis with shorter overall and disease-free survival than those with a high baseline A/G (9). Besides, a lower A/G was an independent risk factor in AIS patients with intravenous thrombolysis for a poor outcome at 3 months (4). To our knowledge, the significance of the A/G in SAP remained unknown.

The present study aimed to explore the association between A/G and SAP, helping physicians identify those AIS patients at high SAP risk and providing more accurate guidance.

## Materials and methods

### Participants

**FIGURE 1:**
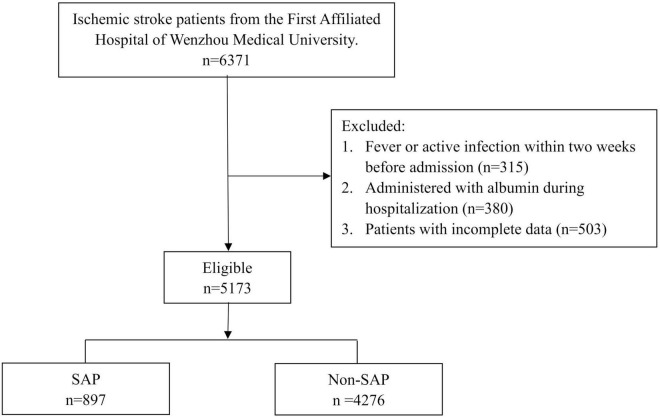
Flow chart of the study. SAP, stroke-associated pneumonia.

As shown in Figure 1, the current investigation is a retrospective analysis conducted at a single site. We reviewed 6,371 patients diagnosed as AIS in the First Affiliated Hospital of Wenzhou Medical University between January 2013 and August 2021. The inclusion criteria were as follows: (1) age 18 years or older; (2) all suspected AIS patients had the diagnosis confirmed by brain computed tomography (CT) scans or brain magnetic resonance imaging (MRI) scans before admission; (3) hospital admission within 7 days after stroke onset.

The exclusion criteria were as follows: (1) fever or active infection within 2 weeks before admission; (2) administered with albumin during hospitalization; (3) patients with incomplete data. Following the inclusion and exclusion criteria, we finally included 5,173 AIS patients, segmented into SAP (*n* = 897) and non-SAP (*n* = 4,276) groups.

The Human Research and Ethics Committee of the First Affiliated Hospital of Wenzhou Medical University approved our retrospective study with the registration number KY2021-R077. The written informed permission requirement was eliminated since all subjects were anonymous. This research adhered to the Declaration of Helsinki and its subsequent revisions.

### Data collection

Sociodemographic and clinical characteristics of AIS patients were obtained from electronic medical records, covering age, gender, body mass index (BMI), hypertension, diabetes, atrial fibrillation (AF), smoking history, drinking history, dysphagia, National Institutes of Health Stroke Scale (NIHSS) score (10), stroke subtype (TOAST classification) (11), and laboratory indicators (leukocyte, creatinine, albumin, and globulin).

Stroke-associated pneumonia was confirmed using the modified Centers for Disease Control and Prevention criteria for hospital-acquired pneumonia during the first week after stroke by combining clinical, radiographic, and laboratory markers of respiratory tract infection. Depending on the conclusions of the dysphagia evaluation, ordinary diets, semi-liquid diet, viscous paste meal, and nasal feeding were prescribed to patients, respectively. The NIHSS score, which reflected stroke severity, was evaluated by at least two neurological physicians upon admission to the neurological ward. The patients with AIS were etiologically classified into large artery atherosclerosis and other subtypes with the trial of ORG 10172 in acute stroke treatment (TOAST) classification criteria. The other subtypes included cardioembolic infarction, small-artery occlusion (lacunae), cardioembolic infarction, stroke of other determined etiology, and stroke of undetermined etiology. Fasting blood samples were collected after overnight fasting on the first morning after admission for all patients. The laboratory parameters, including leukocyte, creatinine, albumin, and globulin, were measured according to standard methods in the clinical laboratory of the First Affiliated Hospital of Wenzhou Medical University.

### Statistical analyses

Baseline data of patient clinical characteristics were descriptively summarized. Continuous variables were presented as median [interquartile (IQR)] or mean ± standard deviation (SD). Categorical variables were presented as frequencies or percentages. These two groups’ comparisons were analyzed by the Mann-Whitney test (for non-normally distributed continuous data), Student’s-test (for normally distributed continuous data), and the chi-square test or the Fisher’s exact test (for categorical variables) as appropriate. The multivariable logistic regression model was constructed to explore the relationship between A/G and SAP, adjusting for the confounders such as age, gender, and NIHSS, and a forest plot was drawn. Patients were divided into four groups according to the number of comorbidities (*n* = 0, 1, 2, and 3), including diabetes, hypertension, and atrial fibrillation. Then, the A/G levels were compared in four groups with Analysis of Variance, and Bonferroni correction was used in the *post hoc* analysis. The threshold of statistical significance level in the current research was defined as *P* < 0.05. R v3.5.1 was used to analyze the research data.

## Results

### Patient’s basic characteristics

Of the 5,173 participants in the final analysis, 17.34% (*n* = 897) were SAP patients and 82.66% (*n* = 4,276) were non-SAP patients. Table 1 summarizes the fundamental research data and clinical features of individuals with AIS. The A/G levels of SAP patients were significantly lower than those of non-SAP patients (1.12 ± 0.22 vs. 1.27 ± 0.23, *P* < 0.001). We found that SAP patients showed elder age, a higher percentage of the male gender, higher BMI, higher NIHSS scores, higher leukocyte counts, higher creatinine, higher globulin, lower albumin and A/G, and were more likely to have a history of AF, and more severe dysphagia (all *P* < 0.05). There were no differences in hypertension, diabetes, smoking, and drinking history between the two groups (all *P* > 0.05).

**TABLE 1:** Characteristics of patients.

Variables	Total (*n* = 5,173)	Non-SAP (*n* = 4,276)	SAP (*n* = 897)	Statistic	*P*
Age	67.77 ± 11.74	66.83 ± 11.68	72.26 ± 10.95	−13.345	<0.001
Gender, n (%)				7.218	0.007
Male	3283 (63.46)	2678 (62.63)	605 (67.45)		
Female	1890 (36.54)	1598 (37.37)	292 (32.55)		
BMI (kg/m^2^)	26.61 ± 4.14	26.45 ± 4.1	27.39 ± 4.25	−6.012	<0.001
Hypertension, n (%)	3629 (70.15)	3009 (70.37)	620 (69.12)	0.495	0.482
Diabetes, n (%)	1414 (27.33)	1173 (27.43)	241 (26.87)	0.092	0.761
Atrial fibrillation, n (%)	827 (15.99)	591 (13.82)	236 (26.31)	85.173	<0.001
Smoking, n (%)	2004 (38.74)	1663 (38.89)	341 (38.02)	0.204	0.651
Drinking, n (%)	1662 (32.13)	1381 (32.3)	281 (31.33)	0.277	0.599
Dysphagia, n (%)				860.697	<0.001
Ordinary diet	2428 (46.94)	2260 (52.85)	168 (18.73)		
Semi-liquid diet	1548 (29.92)	1339 (31.31)	209 (23.3)		
Viscous paste meal	176 (3.4)	144 (3.37)	32 (3.57)		
Nasal feeding	1021 (19.74)	533 (12.46)	488 (54.4)		
NIHSS, median (Q1,Q3)	2 (1, 6)	2 (1, 5)	4 (2, 8)	1353121	<0.001
TOAST, n (%)				21.766	<0.001
Other	3556 (68.74)	2880 (67.35)	676 (75.36)		
Large artery atherosclerosis	1617 (31.26)	1396 (32.65)	221 (24.64)		
Leukocyte (10^9^/L)	7.6 ± 2.37	7.39 ± 2.22	8.62 ± 2.77	−12.492	<0.001
Creatinine (*u*mol/L)	77.65 ± 46.72	76.04 ± 36.06	85.32 ± 79.52	−3.425	<0.001
Albumin (g/L)	36.04 ± 3.93	36.52 ± 3.71	33.79 ± 4.18	18.07	<0.001
Globulin (g/L)	29.6 ± 4.4	29.35 ± 4.34	30.8 ± 4.48	−8.871	<0.001
A/G	1.25 ± 0.23	1.27 ± 0.23	1.12 ± 0.22	18.447	<0.001
A/G quartiles, n (%)				313.197	< 0.001
A/G (≤ 1.09)	1333 (25.77)	906 (21.19)	427 (47.6)		
A/G (1.10–1.24)	1325 (25.61)	1100 (25.72)	225 (25.08)		
A/G (1.25–1.39)	1227 (23.72)	1076 (25.16)	151 (16.83)		
A/G (≥ 1.40)	1288 (24.9)	1194 (27.92)	94 (10.48)		

SAP, stroke-associated pneumonia; BMI, body mass index; NIHSS, National Institutes of Health Stroke Scale; TOAST, the trial of ORG 10172 in acute stroke treatment; A/G, albumin to globulin ratio.

Comorbidities for AIS included diabetes, hypertension, and atrial fibrillation. We further analyzed the relationship between these factors and A/G. Results showed that A/G levels were lower in patients with comorbidities compared with those without comorbidities (hypertension: 1.22 ± 0.23 vs. 1.25 ± 0.23, *P* < 0.001; diabetes: 1.23 ± 0.22 vs. 1.27 ± 0.25, *P* < 0.001; AF: 1.15 ± 0.22 vs. 1.26 ± 0.23, *P* < 0.001). Moreover, we also divided the patients into four groups according to the number of comorbidities (*n* = 0, 1, 2, and 3). Then, the A/G levels were compared in these four groups. The results showed that the A/G ratio declined dramatically with the increase in the number of risk factors (0 vs. 1 vs. 2 vs. 3: 1.30 ± 0.24 vs. 1.25 ± 0.22 vs. 1.21 ± 0.22 vs. 1.11 ± 0.22, *P* < 0.001) (Supplementary Figure 1), which had been adjusted by the Bonferroni’s correction.

### The result of multivariable logistic regression analysis

**FIGURE 2:**
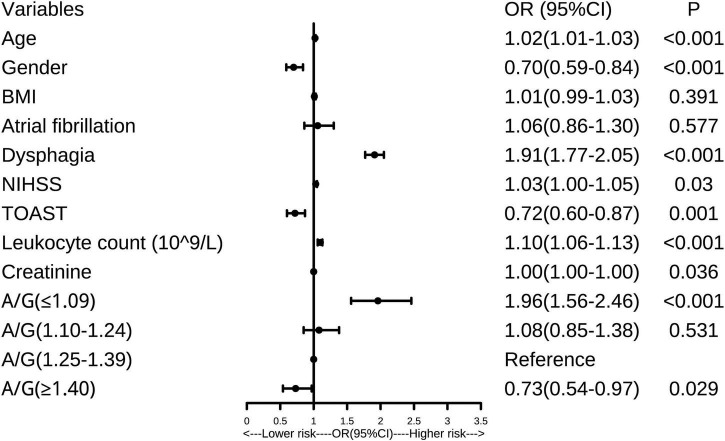
Forest plot of the association between albumin to globulin ratio (A/G) and stroke-associated pneumonia (SAP). A/G, albumin to globulin ratio; SAP, stroke-associated pneumonia; BMI, body mass index; NIHSS, National Institutes of Health Stroke Scale; TOAST, the trial of ORG 10172 in acute stroke treatment; OR, odds ratio.

Multivariate logistic regression analysis was used to determine the risk factors of SAP, and the results are visualized as the forest plot (Figure 2). Based on the quartiles of A/G, AIS patients were divided into the Q1 layer (A/G ≤ 1.09), Q2 layer (A/G = 1.10–1.24), Q3 layer (A/G = 1.25–1.39), and Q4 layer (A/G ≥ 1.4). As shown in Figure 2, compared with the Q3 layer, the Q1 layer (A/G ≤ 1.09) significantly had a higher risk of SAP (OR = 1.96, 95% CI, 1.56–2.46, *P* < 0.001). Interestingly, the Q4 layer (A/G ≥ 1.4) significantly had a lower risk of SAP (OR = 0.73, 95% CI, 0.54–0.97, *P* = 0.029). The results remained significant after adjusting the confounding variables, including age, gender, AF, BMI, dysphagia, NIHSS score, TOAST classification, leukocyte, and creatinine. In addition, females had a lower risk of SAP than males (OR = 0.70, 95% CI, 0.59–0.84, *P* < 0.001). Meanwhile, elder patients (OR = 1.02, 95% CI, 1.01–1.03, *P* < 0.001), more severe dysphagia (OR = 1.91, 95% CI, 1.77–2.05, *P* < 0.001), higher leukocyte counts (OR = 1.10, 95% CI, 1.06–1.13, *P* < 0.001), and higher NHISS scores (OR = 1.03, 95% CI, 1.00–1.05, *P* = 0.03), were all independently associated with a higher risk of SAP. Patients with large artery atherosclerosis showed a lower SAP risk than other types (OR = 0.72, 95% CI, 0.60–0.87, *P* = 0.001). Patients were also divided into the elder (≥ 65) and young (< 65) groups. It was found that the A/G of the elderly was significantly lower than that of the young (1.19 ± 0.21 vs. 1.34 ± 0.23, *P* < 0.001). The role of A/G remained unchanged after adding the age stratifications into the logistic regression model (OR = 2.05, 95% CI, 1.63–2.58, *P* = 0.029).

Moreover, the findings remained significant in both the unadjusted and adjusted models when A/G was added as a continuous variable in the multivariate logistic regression model (all *P* < 0.05). To clarify whether inflammation played a mediating effect on the relationship between A/G and SAP, we further analyzed the three-way association among A/G, leukocytes, and SAP. Firstly, as shown in Table 1, patients with SAP had lower A/G levels and higher leukocytes (all *P* < 0.001) than the non-SAP group. Secondly, lower A/G levels were linked to higher leukocytes (*r* = −0.168, *P* < 0.001). Given the above, the leukocytes might play a mediating role between A/G and SAP.

## Discussion

Clinically, SAP significantly aggravates the conditions of AIS patients, increases the occurrence of severe disabilities, and intensifies the risk of death (12). Thus, it is essential to early identify AIS patients who may easily suffer from SAP and then schedule individualized treatment regimens to reduce the risk of SAP. To our knowledge, no prior research has examined the relationship between serum A/G and SAP risk in AIS patients. Our findings established a substantial correlation between A/G and SAP. Patients with a lower A/G (A/G ≤ 1.09) had a higher SAP risk, whereas those with a higher A/G (A/G ≥ 1.4) had a lower SAP risk. A/G can be considered an effective predictor for SAP in AIS patients.

Albumin to globulin ratio (A/G) includes serum albumin and globulin, two objective and easily measurable components of human serum proteins commonly employed in clinical practice to reflect and identify inflammation and malnutrition (4). Recently, some studies have shown that higher A/G may protect patients from various ailments. For example, research on the elderly revealed a substantial positive relationship between cognitive performance and the A/G ratio. Better cognitive function was associated with a high serum A/G in community-dwelling older people (13). Patients with periprosthetic joint infection, a severe complication of total joint arthroplasty, exhibited a significantly lower A/G ratio than aseptic patients (7). According to previous publications, A/G was independently associated with various cancers, such as lung cancer, squamous cell carcinoma of the maxillary sinus, diffuse large B cell lymphoma, and metastatic gastric cancer. These studies concluded that cancer patients with a low A/G had a worse overall and disease-free survival rate than those with a higher A/G (9, 14–17). The present study indicated that patients with a lower A/G (A/G ≤ 1.09) had a higher SAP risk, while those with a higher A/G (A/G ≥ 1.4) had a lower risk.

Since inflammation and malnutrition significantly influence the occurrence of SAP, we speculated that the association between A/G and SAP might be explained in the following ways. The first possibility is that low A/G reflects malnutrition, and poor nutritional status is a vital risk factor in the course of SAP. The AHA/ASA Guidelines for the early management of AIS patients recommend early attention should be given to AIS patients at risk of malnutrition (18). After AIS, malnourished patients are more likely to develop pneumonia or other kinds of infection during hospitalization (12). The second possibility is inflammation plays a role in SAP. In the acute phase of stroke, inflammatory factors enter the systemic circulation through the damaged blood-brain barrier, leading to a systemic inflammatory response (19). A prospective study investigated the effects of long-term treatment with exogenous supplemental albumin and found that albumin reduced the levels of inflammatory-related cytokines such as interleukin-6 (IL-6), interleukin-1 (IL-1) receptor antagonist, vascular endothelial growth factor (VEGF), and granulocyte colony-stimulating factor (G-CSF) (20). Furthermore, an *in vitro* experiment described albumin added to the albumin dialysis reduced the measurable amount of proinflammatory cytokine levels of IL-6, indicating albumin may also be an anti-inflammatory factor (21). Serum globulin, a classic inflammatory factor, increases during the inflammatory process (22). Inflammation typically causes an increase in the leukocytes. This study also found that leukocytes might serve a mediating effect between A/G and SAP, which further confirmed that inflammatory mechanisms might play an essential role between A/G and SAP. A third possibility is that high levels of albumin and low levels of globulin have neuroprotective effects, which may influence the risk of SAP by detoxifying endogenous and exogenous molecules, maintaining the integrity of the blood-brain barrier, increasing hypoxia tolerance, decreasing brain edema, and modulating neural apoptosis and inflammation reactions (23–25).

Comorbidities for AIS include diabetes, hypertension, and atrial fibrillation. We further analyzed the relationship between these factors and A/G. Results showed that A/G levels were lower in patients with comorbidities than those without these issues. This finding provided evidence that nutritional status might be associated with the comorbidities or risk factors for AIS, and it might be meaningful to improve the nutritional status of stroke patients. Besides, we also found that the A/G ratio declined dramatically with the increase in the number of risk factors, which highlighted the importance of the A/G in AIS and its complications. Based on this finding, we will further explore the link between A/G and AIS in the future.

This research also discovered that males, advanced age, more severe dysphagia, and a higher leukocyte count were all related to an increased SAP risk in patients with AIS, consistent with previous reports (26, 27).

Several limitations should be addressed in our investigation. (1) this study was retrospectively designed, which may not accurately reflect the causation relationship between A/G and SAP; (2) the patients came from a single medical center, so the validation of our findings required multi-center research with a bigger sample size; (3) we only analyzed a single measurement of baseline A/G on admission and did not consider changes in A/G during hospitalization or over the follow-up period; (4) This study did not collect anthropometric indicators such as arm circumference and waist-to-hip ratio. In the future, we will further improve the research design and add more anthropometric indicators mentioned above, which could help us better explore the effect of nutritional status on SAP.

## Conclusion

In conclusion, statistically, a lower A/G level is associated with a higher SAP risk. The findings underscored the critical role of A/G in predicting SAP in patients with AIS and stressed the necessity to uphold more appropriate measures to minimize the incidence of SAP in patients with a lower A/G. Future research should investigate the effect of nutritional intervention to improve the A/G ratio on the outcome of AIS and SAP, which will be of great importance.

## Data availability statement

The original contributions presented in this study are included in the article/Supplementary material, further inquiries can be directed to the corresponding authors.

## Ethics statement

The Human Research and Ethics Committee of The First Affiliated Hospital of Wenzhou Medical University approved our retrospective study with the registration number: KY2021-R077. The written informed permission requirement was eliminated since all subjects were anonymous. This research adhered to the Declaration of Helsinki and its subsequent revisions.

## Author contributions

YL and WR contributed to the conception and design of the research. WR performing statistical analysis. LC contributed to the manuscript draft and performed the analysis with constructive discussions. MX, WR, and QH contributed to the collection of the data. All authors critical revision of the manuscript and approved the submitted version.

## Supplementary material

Supplementary Figure 1 Compare the albumin to globulin ratio (A/G) ratio in four groups. Zero, one, two, and three represent the numbers of comorbidities of AIS, including hypertension, diabetes, and atrial fibrillation. The A/G levels were compared in these four groups. A/G, albumin to globulin ratio. ****P* < 0.001.
