# Supplementary figures and images for: Clinical significance of albumin to globulin ratio among patients with stroke-associated pneumonia
Source: Front Nutr. 2022 Aug 16;9:970573. doi: 10.3389/fnut.2022.970573 (PMC9424928; doi:10.3389/fnut.2022.970573)

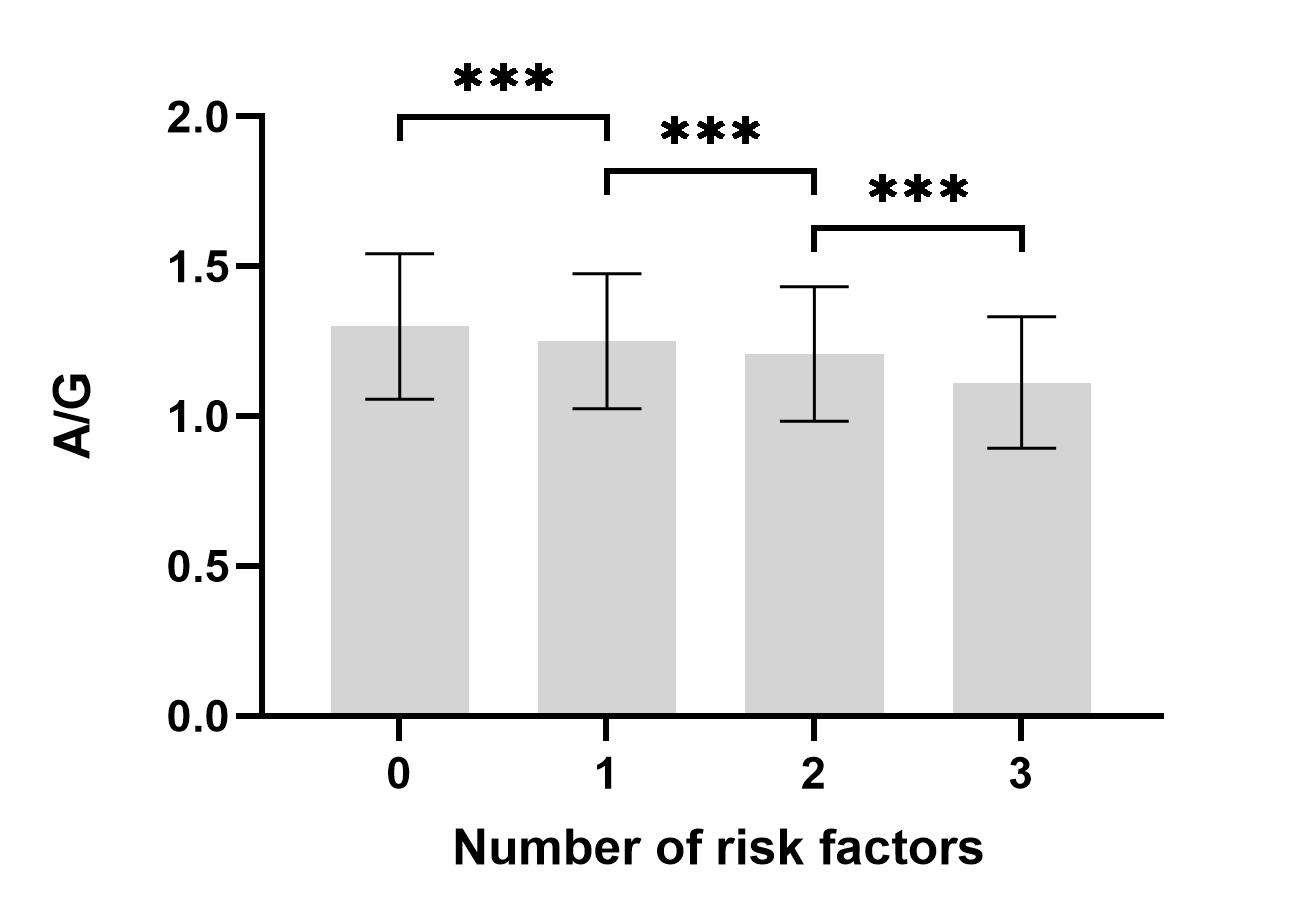

Supplement: Supplementary Figure 1 — Compare the albumin to globulin ratio (A/G) ratio in four groups. Zero, one, two, and three represent the numbers of comorbidities of AIS, including hypertension, diabetes, and atrial fibrillation. The A/G levels were compared in these four groups. A/G, albumin to globulin ratio. ***P < 0.001. [file Image_1.jpeg]
